# The Study of the Face as an Index of the Brain

**Published:** 1883-03

**Authors:** F. Warner


### ARTICLE IV.

### *The Study of the Face as an Index of the Brain.*

Dr. Francis Warner read a paper on the above before the Section of Medicine of the British Medical Association, which possesses much interest.

The face is a region of the body well worthy of clinical study. We may observe its form, color, and mobility and the effects of movement in causing expression. The movement is the outcome of the action of the brain.

The tissues forming the structure which we call the face are mainly the skin, with its vessels and vaso-motor system; the subcutaneous fat; the facial muscles, supplied by the facial nerve, and some of the masticatory muscles, supplied by the fifth nerve.

Most of the variations of facial expression are produced by the facial muscles, which are acted upon by the changes in the brain; and these are the special indications of the cerebral condition to which attention will here be called. The muscles of mastication are less expressive of the condition of the brain than are the facial muscles; they may become the subject of spasm, atrophy (see Case, *Lancet*, January 7th, 1882), or may produce teeth-grinding, as the result of conditions of the brain.

In almost all cases, the best indications of the conditions of the central nerve mechanism are its effects as seen in the spontaneous action of the muscles; and it is by the results that we usually judge of the central condition. Such effects are conveniently spoken of as nerve-muscular signs. Nerve-muscular signs are the best indices of the brain; the change in the brain affects the muscles; these control the position of the visible parts; and, from the facial changes resulting, we gather our information.

We are here studying the results of cerebral action upon muscles, according to well understood principles of physiology; while physiognomy deals mainly with the shape of the brain-case, and the passive condition of the face.

The principal movements of the facial muscles are these:

1. Dilatation and contraction of the facial foramina; probably this corresponds in significance to flexion and extension.

2. Elevation and depression of parts. Such conditions are well seen by comparing the two sides of the face in a case of Bell's paralysis.

3. Retraction and drawing forward of parts, as in grinning and screwing up the mouth.

To examine a face, hold a piece of paper in front of it, with one edge vertical; either half of the face can then be covered in turn. Again the face may be divided into three zones, by holding the paper with one margin horizontal, leaving the forehead above the eyebrows uncovered; or the face below the lower margin of the orbits may alone be exposed, showing the mouth, most of the cheeks, and the alæ nasi; or, again, the middle zone, including the eyes with the upper and lower eyelids, may be viewed alone.

After looking for symmetry in a face, the nerve-muscular condition of the individual parts may be compared. A different condition of the different zones has, possibly, about the same kind of significance as an unequal condition of flexion and extension of the different fingers. A condition of different activity in the three zones of the face is a departure from physiological calmness (*e. g.*, a smile, or snarl); it may be normal or abnormal. An unequal condition of the different zones is very common in "nervous people."

In the upper zone, we have the occipito frontalis and the corrugator supercilii. Here we see the outcome of brain action in those conditions termed grief, surprise, etc., producing muscular action and corrugating the forehead. The occipito frontales are often seen overacting in imbeciles, and in cases of chorea; sometimes, also, they overact as a mere "chronic nervous habit." Symmetry in this zone is usually maintained. In the middle zone asymmetry is less uncommon, but is seen in winking and in ptosis. The important muscles here are the orbiculares oculorum. With megrim, and in conditions of depression, a marked change is often seen in the midfacial zone due to a fulness about the eyes, especially about the under eyelids: the orbiculares have lost their tone; the skin hangs too loosely; the skin of the lower eyelid, instead of forming a convex surface, passes as a plane from the ciliary margin to the lower margin of the orbit. If the patient be made to laugh, the orbiculares are energized for the moment, and the look of depression is lost.

Passing on to the consideration of the lower facial zone, we see here the most marked effect of facial spasm or palsy from brain-disease—*e. g.*, in hemiplegia and lesion of the crus cerebri. Thus the weakness of the face is well demonstrated by making the patient show his teeth or smile. Now, the muscles of this region are those most commonly seen in spontaneous action in imbeciles; it is these muscles that work awkwardly in nervous one-sided grinning, and in this region we most commonly see asymmetry of the features, due to nerve-muscular conditions. It is interesting to note that this region of the face is the most affected by brain-disease (paralysis), and in "nervousness" (irregularity of the mobile features). Again, it is the levator labii superiors in the lower zone which produces one-sided snarling one of the lowest expressions produced by the human face. In complete development and perfect health the features are usually regular in passive form and in symmetry of movement. In most expressions, the symmetry of bilateral movement is complete; from this we infer that the nerve-mechanisms for each side of the face are intimately connected.

In observing how easily facial asymmetry is brought about, we see evidence that the union of the facial centress is easily dissolved and not very strong, the asymmetry being especially seen in the lower zone; thus asymmetry is produced by nervousness (see one-sided grinning), by the desire to attack (see *Darwin on the Expression of the Emotions*, page 250,) (snarling), or by such defective nutrition or development as produces unequal features when in motion. The higher, more intelligent expressions, are symmetrical. Union of the facial centres is less perfect than the union between the motor centres of the eyes for the cerebral condition producing "emotion" cannot cause disassociated movement of the eyes. We find here an illustration that symmetry of action as well as symmmetry of structure, is part of the law of beauty.

It may be remarked as a peculiarity of the facial muscles in man, that they are usually free or disengaged, not mainly occupied in doing a definite work, but their movements are mainly the spontaneous outcome of brain-action. The facial muscles are usually very mobile, and often illustrate the struggle of nerve-mascular actions; this may be seen in the conflict of the muscles about the mouth in the endeavor not to cry. The study of nerve muscular signs in the limbs shows the importance of observing whether "the movement of small parts"—*e. g.* the fingers—be thoroughly good; this kind of observation in the facial region is, I think represented by "the finer movements of expression." These are totally absent in some cases of paralysis agitans, although the larger movements of the face can be voluntarily performed. When fatigued, the brain does not act well in producing the small, fine movements of either hand or face. We sometimes see a nervous play of the features: this, I imagine, depends upon slight irregularity of the muscles producing slight movements. In convulsion, both clonic and tonic, and in tetanus, the larger muscles usually produce the most marked effect.

In observing different types of face, we become at once struck with the fact that some faces express intellectuality, others vulgarity; some faces are very mobile and very expressive, others are passive and immobile.

The peculiarity of vulgar faces may be roughly divided into two elements: 1. Physiognomy or the shape of the brain-case and face, together with the character of the facial tissues, and the structure of the features and parts of the face. Here, probably, we have an example of the co-incident defective or coarse development of the face and the brain. This illustrates the relation of morphology and function, the structure of the face, and the coincident structure of the brain which moves the face. Elements contributing to this character of face area large under jaw, a thick immobile condition of the facial skin, thick lips, etc. It will be seen that these are mainly conditions in the development increasing the protective character of the skin of the face; the thick immobile tissue is better able to resist the action of external agencies, but it is also less mobile under the action of nerve-muscular changes. The large lower jaw may be very useful for mastication or defence, but it does not serve to increase the play of nerve muscular actions.

2. The second typical characteristic is the nerve-muscular condition of the face. Such signs are more directly indicative of intellectuality of the brain; hence we should study a face as the index of the brain, when it is seen in action as well as when at rest. The mobility in the different zones and the relative condition of the mental nerve-mechanism. These considerations afford some evidence that facial expression results from nerve-muscular action, the outcome of the motor action of that part of the nerve-mechanism which produces mental states.—*Medical and Surgical Reporter.*

